# Habitat Association and Conservation Implications of Endangered Francois’ Langur (*Trachypithecus francoisi*)

**DOI:** 10.1371/journal.pone.0075661

**Published:** 2013-10-09

**Authors:** Yajie Zeng, Jiliang Xu, Yong Wang, Chunfa Zhou

**Affiliations:** 1 College of Nature Conservation, Beijing Forestry University, Beijing, China; 2 Center for Forestry, Ecology, and Wildlife, School of Agriculture and Environmental Science, Alabama Agricultural and Mechanical University, Normal, Alabama, United States of America; 3 Ministry of Education Key Laboratory for Biodiversity Science and Ecological Engineering, College of Life Science, Beijing Normal University, Beijing, China; 4 College of Forest Resources and Environment, Nanjing Forestry University, Nanjing, China; Institut Pluridisciplinaire Hubert Curien, France

## Abstract

Francois’ langur (*Trachypithecus francoisi*) is an endangered primate and endemic to the limestone forests of the tropical and subtropical zone of northern Vietnam and South-west China with a population of about 2,000 individuals. Conservation efforts are hampered by limited knowledge of habitat preference in its main distribution area. We surveyed the distribution of Francois’ langur and modeled the relationship between the probability of use and habitat features in Mayanghe National Nature Reserve, Guizhou, China. The main objectives of this study were to provide quantitative information on habitat preference, estimating the availability of suitable habitat, and providing management guidelines for the effective conservation of this species. By comparing 92 used locations with habitat available in the reserve, we found that Francois’ langur was mainly distributed along valleys and proportionally, used bamboo forests and mixed conifer-broadleaf forests more than their availability, whereas they tended to avoid shrubby areas and coniferous forests. The langur tended to occur at sites with lower elevation, steeper slope, higher tree canopy density, and a close distance to roads and water. The habitat occupancy probability was best modeled by vegetation type, vegetation coverage, elevation, slope degree, distances to nearest water, paved road, and farmland edge. The suitable habitat in this reserve concentrated in valleys and accounted for about 25% of the total reserve area. Our results showed that Francois’ langur was not only restricted at the landscapes level at the regions with karst topography, limestone cliffs, and caves, but it also showed habitat preference at the local scale. Therefore, the protection and restoration of the langur preferred habitats such as mixed conifer-broadleaf forests are important and urgent for the conservation of this declining species.

## Introduction

Effective management of wildlife populations to conserve threatened species or promote biodiversity generally entails habitat management based on the understanding of species’ needs [1]. Many wildlife species such as primates are threatened by human activities such as urban sprawl and resource exploitation, which lead to deforestation, forest fragmentation, and habitat loss [2]. Habitat fragmentation and loss are the major threats to karst forest dwelling langur species [3]–[5], and different primates might have varying responses to changes in habitat availability and quality (e.g. [6]). In addition to the direct impact to primate fitness such as increased mortality, the habitat loss or changes can cause changes in primates’ behavior, for example, the white-headed langur (*Presbytis luecocephalus*) spend less time feeding in high quality habitat than they do in low quality habitat [7]. Therefore, studies of habitat use or habitat suitability have been an important aspect of primate ecology and primate conservation [8].

Francois’ langur (*Trachypithecus francoisi*) is an endangered primate that is endemic to the limestone forests of the tropical and subtropical zones of northern Vietnam and South-west China [9], [10], with a current estimated global population of about 2,000 individuals [6]. Due to forest loss, some populations have been locally extirpated from some parts of their historical ranges [11]. For instance, this species has been extirpated from five of its original distribution areas in Guizhou, and has become restricted to only five isolated sites [5]. Although this species is now listed as endangered on the International Union for Conservation of Nature (IUCN) Red List, and as a first-grade protected species under wildlife laws in China, little is known about its habitat use and factors affecting habitat selection or association except for some sleeping site selection and feeding habitat use (see [6], [12]).

As one of the five remaining sites in Guizhou, China, Mayanghe National Nature Reserve is in the northeastern part of the Francois’s langur range and holds the highest sub-population of this species [5]. A survey in 2003 estimated that there were 78 groups with about 589 to 714 individuals (Management Bureau of Mayanghe National Nature Reserve, unpublished data). A survey conducted by Fauna & Flora International (FFI) in 2004 recorded 59∼62 groups with about 426 to 442 individuals in the same reserve (Luo Yang, unpublished data). These numbers indicate the reserve holds an estimated one-third of the species’ global population [13]. Thus, the reserve is of global conservation importance. Although the decrease in the number of langurs between the two surveys could be due to variations in sampling intensity and methods, the population of this species in the reserve is increasingly threatened by factors such as habitat disturbance and loss [13].

We studied habitat use of Francois’ langur in Mayanghe National Nature Reserve in 2008 and 2009 with the main objectives of 1) deriving a better understand of habitat requirement and use of Francois’ langur, 2) developing models for predicting the habitat availability and distribution of Francois’ langur, and 3) providing quantitative information for the effective conservation and management of this threatened species’ habitat.

## Materials and Methods

### Ethics Statement

The study was approved by Ministry of Science and Technology of People’s Republic China and State Forestry Administration. The specific permission for surveying the Francois’ langur in Mayanghe National Nature Reserve was also granted by the State Forestry Administration, the local forestry department, and Management Bureau of this reserve. Our study protocol involved searches on-foot for the calls, individual sightings, or the fresh fecal traces using audial or visual signs through ears or binoculars. We always maintained a distance from the langurs and other animals to minimize potential disturbance to them and protect the accuracy of the data collected.

### Study Area

**Figure 1:**
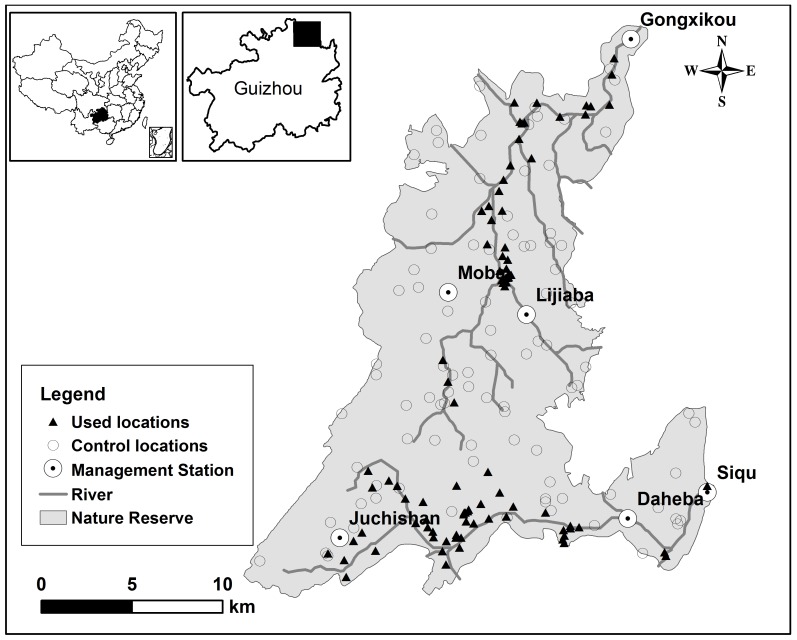
Francois’s langur detected (used) and random (control) locations in Mayanghe National Nature Reserve, China. Used locations included the locations from line transects and targeted surveys based on the reports of rangers; control locations were randomly generated using ArcGIS 9.2; a Management Station managed and monitored a management and monitoring sub-units.

We conducted the study in Mayanghe National Nature Reserve (28°37′30″∼28°54′20″N, 108°3′58″∼108°19′45″E) in the subtropical zone of northeastern Guizhou Province, China (Figure 1). This reserve occupies an area of 31,113 ha and is divided into six management and monitoring sub-units; each sub-unit is managed and monitored by a Management Station (Figure 1). This reserve has the karst geophysical features with an altitude ranging from 280 m to 1441 m; the climate is warm, humid and rainy. Mean annual temperature is 18.3°C, and mean annual precipitation is 1139 mm [14]. The natural vegetation of the reserve is characterized by evergreen broadleaf forest, mixed evergreen-deciduous broadleaf forest, and mixed conifer-broadleaf forests with forest cover being about 64% [14]. Mayanghe River and Hongduhe River flow through this reserve, and the vegetation along the river valley is mainly evergreen broadleaf forest. Francois’ langur and Rhesus Macaque (*Macaca mulatta*) are the only two nonhuman primates living at this reserve [6].

### Surveying Francois’ Langur

We surveyed the occurrence and habitat association of the Francois’ langur across the entire reserve in October 2008 and between July and August 2009. Because of the difficult terrain of limestone mountain topography, we were not able to allocate survey transect randomly or evenly across the space [5]. We employed a sampling approach similar to a previous study [13] by first divided the reserve into six relatively similar size units according to the six management sub-units, and we then established two transects within each unit. Each transect was approximately 4 km in length and 0.1∼0.15 km wide each side. Some existing trails were used when the transects were too difficult to access [6], [13], [15]. We determined starting point initially, and then the direction of each transect randomly based on a grid system overlaid on each sub-unit, but conditioned on covering all vegetation types in each unit. The total transect length across the study area was about 48 km. In addition to transect surveys, we established 4∼5 observation stations on higher ground along each transect. The observers at these stations scanned with binoculars and listened for langur vocalizations, and searched the cliffs along the transect for fresh fecal-traces of langurs when no langurs were detected. We carried out surveys during the daytime from 6∶00 to 19∶00 with the help of the two local guides with extensive experience in the study area and who had participated in previous Francois’ langur surveys in this reserve. Each transect was surveyed at least twice during the study.

To cover the habitat of each management sub-unit that could not be surveyed with transects due to the steep topography, we conducted targeted surveys to confirm the presence of Francois’ langur, based on the reports of 17 rangers of the six stations of the reserve to complement the line-transect surveys. The rangers were well-trained for identifying langur and other primates and animals, and they patrolled their sections of the reserve at least once a month. The combination of our transect surveys and spot-checked locations based on rangers’ reports covered the majority of the reserve and all habitat types.

We marked each direct observation and each fresh fecal trace of Francois’ langur from transects or targeted surveys on a topographic map (1∶10000). Additionally, we recorded group characteristics such as individual numbers, juveniles, or adults.

### Habitat Quantification

To measure the habitat characteristics in the study area, we used a 1∶10000 vegetation map supplied by the Management Bureau of the reserve and a forest resources inventory of 2006 to produce a digital land-cover map of the study area. The inventory of forest resources were obtained by field surveys organized by the local forestry department in 2005 and 2006. During those surveys, the habitat in this reserve was divided into many parcels or sub-compartments. The vegetation in each sub-compartment was ground-truthed according to a standard protocol. The habitats within the study area were classified according to plant species presence, species composition, and the proportions each species occupied in each sub-compartment of the reserve [6]. We gound-truthed this dataset in the field; the accuracy of the habitat identification and classification was greater than 90%. We also used a digitized 1: 50000 topographic map supplied by Institute of Geography Sciences Research and a 1: 10000 topographic map provided by the Management Bureau of the reserve to extract the landscape variables.

We used ArcGIS 9.2 (Environmental Systems Research Institute) to generate 13 variables (Table 1) that were potentially important for Francois’ langur habitat use based on our field observations and previous researches (e.g. [15], [16]; Wang Shuangling, unpublished data). These variables belonged to three groups: 1) vegetation (V), 2) terrain (T), and 3) distance (D). The variables in V reflected vegetation features [15], [16], variables in T were selected based on topographic features found to be important in previous studies [13], [15], [16], and variables in D represented mean distances to human disturbance or water availability [6], [16].

**Table 1:** Habitat variables, classes, and their definitions of Mayanghe National Nature Reserve, China.

Class	Variable	Description
Vegetation (V)	Vegetation type (VET)	Vegetation types classified based on the land-cover map, including farmland, evergreen broadleaf forest, mixed evergreen-deciduous broadleaf forest, shrub, bamboo forest, deciduous broadleaf forest, coniferous forest, and mixed conifer-broadleaf forest.
	Tree canopy density (TCD) (%)	Measured as the proportion of the ground covered by the projection of the tree canopy
	Vegetation comprehensive coverage (VCC) (%)	Measured as the proportion of the ground covered by the projection of all vegetation
Terrain (T)	Elevation (ELE) (m)	Elevation of the locations above sea level
	Slope position (POS)	The position on the slope, classified into the upper 1/3 part of the slope and the ridge, the middle 1/3 part of the slope, and the lower 1/3 part of the slope and the valley
	Slope degree (SLO) (°)	Degree of the slope at the locations ranging from 0° to 90°
	Slope aspect (SLA) (°)	Aspect of the slope at the locations ranging from 0°to 360°
Distance (D)	Distance to residence (DNR) (m)	Distance from the locations to the nearest edge of residence area
	Distance to water (DWA) (m)	Distance from the locations to the nearest water body
	Distance to farmland (DFA) (m)	Distance from the locations to the nearest edge of farmland
	Distance to paved road (DPA) (m)	Distance from the locations to the nearest pave road
	Distance to unpaved road (DUPA) (m)	Distance from the locations to the nearest unpaved road
	Distance to lane road (DLA) (m)	Distance from the locations to the nearest lane road

### Data Analysis

To reduce the probability of inter-dependence among the adjacent observations, we excluded locations for further analysis if the distance between each was <800 m unless we were certain the adjacent locations were used by different langur groups (based on characteristics such as group size, group structures, and morphological features of some individuals). We used the sine- and cosine- transformed value of the slope aspect [17] and kept them together for further analysis. We used independent t test to examine if the habitat variables differed between those from line transects and those from spot-checked locations before we merged them. We employed a used-available design to evaluate habitat use and selection [18], [19] by langurs. We randomly generated the same number of locations (hereafter “control locations”) as the detected locations (hereafter “used locations”) with the reserve boundaries as our available habitat limit using ArcGIS 9.2. Because our sample size was relatively large (>30, see below) and the same for the used and control locations, we used two independent samples t-test to examine if the individual numerical habitat variables differed between used and control locations [17]. For categorical variables, we used χ^2^ goodness-of-fit test and Bonferroni Z-statistic to determine whether the use of different habitat categories by langurs was proportional to the availability [20]. We considered the proportion of the used locations within a given habitat type as used, and the proportion of habitat types in the entire reserve as available.

Since our response variable was binary, we used logistic regression [21], [22] to model the relative probability of a location being used as a function of habitat variations [18], [22], [23]. We first built univariate logistic regression models using variables that showed significant difference between used and random points from the t-test above. We retained habitat variables that were potentially important based on the univariate logistic regression analysis (P<0.25) [22] for multivariate logistic models. Categorical variables (vegetation type and slope position) were dummy coded for these analyses.

Using the retained variables, we built seven hierarchical groups of multivariate logistic models that included 1) vegetation variables (V), 2) terrain variables (T), 3) distance or anthropogenic features (D), 4) a combination of V and T, 5) a combination of V and D, 6) a combination of D and T, and 7) a combination of all above. In each of these seven group process, if variables were correlated (|r|>0.6), we tested multiple sub-models using different combination of variables so that variables in the same models had low correlation (Table 2).

**Table 2:** Mean and standard deviation of habitat variables measured at used and random (control) locations of Francois’s langur in Mayanghe Nature Reserve, China.

Variables	Used points(n = 92)	Controlpoints(n = 92)	t-value	df	p-value
ELE(m)	639.4±210.1	837.1±229.2	−6.1	182	<0.01**
SLO( °)	43.4±12.7	23.2±13.1	10.7	182	<0.01**
TCD(%)	52.2±23.0	41.3±29.5	2.8	172	<0.01**
VCC(%)	78.9±17.7	66.4±21.6	2.2	175	0.03*
SIN	−0.1±0.7	0.1±0.7	−1.0	182	0.30 ns
COS	0.1±0.7	0.1±0.7	0.2	182	0.87 ns
DRE(m)	1907.5±1119.6	1808.5±1024.4	0.6	182	0.53 ns
DWA(m)	371.6±391.2	895.7±917.5	−5.0	123	<0.01**
DFA(m)	204.1±218.7	139.5±207.8	2.0	182	0.04*
DPA(m)	2191.2±1862.1	3728.2±2389.3	−4.9	172	<0.01**
DUPA(m)	4374.4±2600.6	3638.6±2968.0	1.8	182	0.08 ns
DLA(m)	252.1±254.6	217.0±244.9	0.9	182	0.34 ns

The abbreviations of the variables were described in Table 1. SIN and COS are the sine- and cosine-transformations of the aspect, respectively. **P<0.01, *P<0.05, “ns” = not significant. The degree of freedom of t-test varied by different variables because of the adjustment for the equal variance [17].

We employed information-theoretic approach to compare the candidate models for explaining habitat use [24]–[26]. Because the number of parameters of some models was large relative to sample size, we ranked models according to the second order Akaike’s Information Criterion (*AICc*, see [24]) which provided better assessment with small sample size. We calculated △*AICc*, *AICc* weights (*w_i_*), deviance (−2 log likelihood), and Nagelkerke R^2^ to provide additional measures of model fit [24]. We considered the model with the highest *w_i_* as the best model [24] and used the area under curve (AUC) of the receiver operating characteristic (ROC), overall classification accuracy, and Nagelkerke R^2^ to assess the fit of the best model [27]. We then used the model to map the probability of habitat use using ArcGIS 9.2 for the entire reserve surveyed. Locations with predicted use probability above average use probability estimated across the entire reserve (threshold) were classified as suitable habitat [28], [29]. For all analyses, we reported mean with standard deviation, and determined statistical significance at α level of 0.05 unless otherwise indicated.

## Results

Mayanghe National Nature Reserve was composed of eight habitat types, including shrub (33.9%), farmland (30.4%), coniferous forest (16.4%), deciduous broadleaf forest (6.1%), mixed conifer-broadleaf forest (4.8%), mixed evergreen-deciduous broadleaf forest (4.6%), evergreen broadleaf forest (3.4%), and bamboo forest (0.4%).

**Figure 2:**
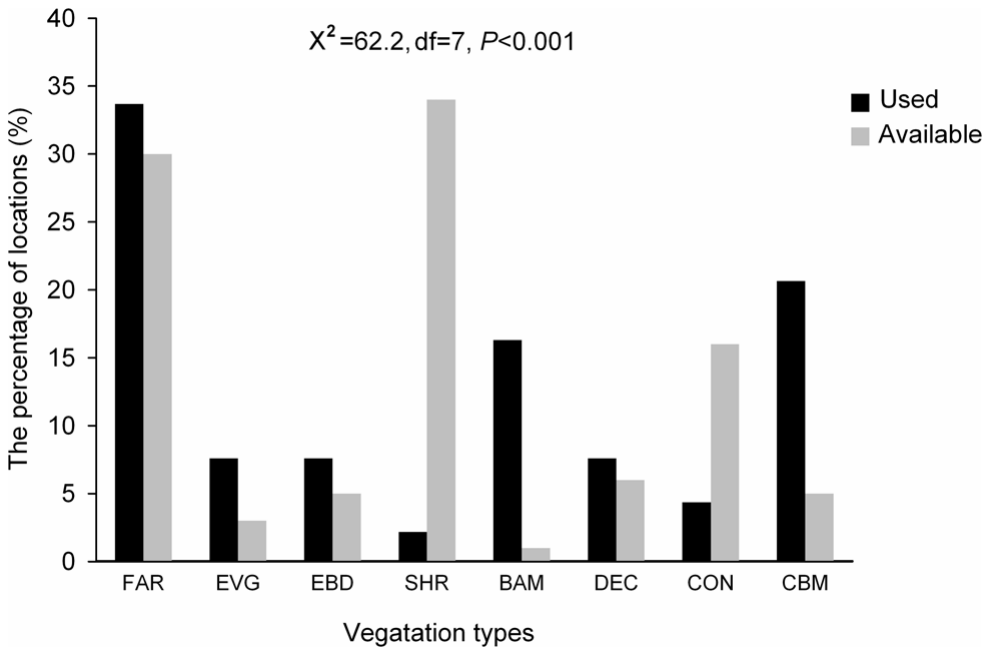
Habitat preference of Francois’ langur based on vegetation types of detected locations and availability in Mayanghe National Nature Reserve, China. FAR = Farmland, EVG = Evergreen broadleaf forest, EBD = Mixed evergreen-deciduous broadleaf forest; SHR = Shrubby area; BAM = Bamboo forests, DEC = Deciduous broadleaf forest; CON = Coniferous forest, and CBM = Mixed conifer-broadleaf forests.

We detected 92 used locations including 64 used locations from transect surveys and 28 confirmed locations reported by the reserve rangers. We did not detect any difference for variables we measured between these two sources (t test, all P>0.05). Francois’ langur showed preferences to different vegetation types (Figure 2, χ^2^ = 62.2, df = 7, P<0.001). Generally, the langurs tended to use bamboo forest and mixed conifer-broadleaf forests more than available (χ^2^ test, P<0.001), and tended to avoid shrubby area and coniferous forest (χ^2^ test, P<0.001). The sites used by the langurs tended to have lower elevation, steeper slope, higher tree canopy density, higher vegetation coverage, and a closer distance to water and paved road, however they tended to keep a distance from the edge of the nearest farmland. We did not detect differences between used and control locations in relation to slope aspect, and distances to the nearest residence, unpaved road and lane road (Table 2). Two pairs of variables, vegetation type (VET) with Tree canopy density (TCD) and Vegetation comprehensive coverage (VCC) with TCD, had relatively high correlation (|r|>0.6). Therefore, we tested 11 candidate models of possible variable combinations, including 2 models with V group, 1 model with T group, 1 model with D group, 2 models with V and T groups, 2 models with V and D groups, 1 model with T and D groups, and 2 models with V+T+D groups (Table 3). Of the three single group models (V, T, and D), the terrain variables (R^2^ = 0.66) had a much stronger influence on habitat selection of langurs than did vegetation and distance variables (Table 3). Among the vegetation variables, vegetation type and vegetation coverage (R^2^ = 0.19) had a stronger effect on habitat selection than did tree canopy density (R^2^ = 0.05, Table 3). The best model included the following variables: vegetation type, vegetation coverage, elevation, slope degree, distances to the nearest water, paved road and the edge of farmland (*w*_i_ = 0.99, AUC = 0.95, Nagelkerke R^2^ = 0.73, 87% prediction accuracy, Table 3).

**Table 3:** Candidate logistic regression models with number of parameters (K), second-order Akaike’s Information Criterion (*AICc*), difference *of AICc* from the lowest *AICc* (Δ*AICc*), and model weight (*w_i_*) for estimating the probability of habitat use of Francois’s langur in Mayanghe Nature Reserve, China.

Model	Variables in the model	k	R^2^	−2 log likelihood	*AICc*	Δ*AICc*	w_i_
V	TCD	3	0.05	247.4	247.7	137.0	<0.01
	VET+VCC	4	0.19	226.3	226.8	116.2	<0.01
T	ELE+SLO	4	0.66	128.9	129.3	18.6	<0.01
D	DWA+DFA+DPA	5	0.35	199.9	200.6	89.9	<0.01
V+T	VET+VCC+ELE+SLO	6	0.70	118.8	119.8	9.1	0.01
	TCD+ELE+SLO	5	0.66	128.7	129.4	18.7	<0.01
V+D	TCD+ DWA+DFA+DPA	6	0.35	198.2	199.2	88.6	<0.01
	VET+VCC+DWA+DPA+DFA	5	0.43	182.8	183.5	72.8	<0.01
T+D	ELE+SLO+DWA+DFA+DPA	7	0.69	122.1	123.4	12.7	<0.01
V+T+D	VET+VCC+ELE+SLO+ DWA+DPA+DFA	9	0.73	108.5	110.7	0	0.99
	TCD+ELE+SLO+DWA+DPA+DFA	8	0.69	121.4	123.2	12.4	<0.01

The habitat variables were classified to three groups: 1) vegetation (V), 2) terrain (T), and 3) distance (D). See Table 1 for the descriptions of variables and their abbreviations.

**Figure 3:**
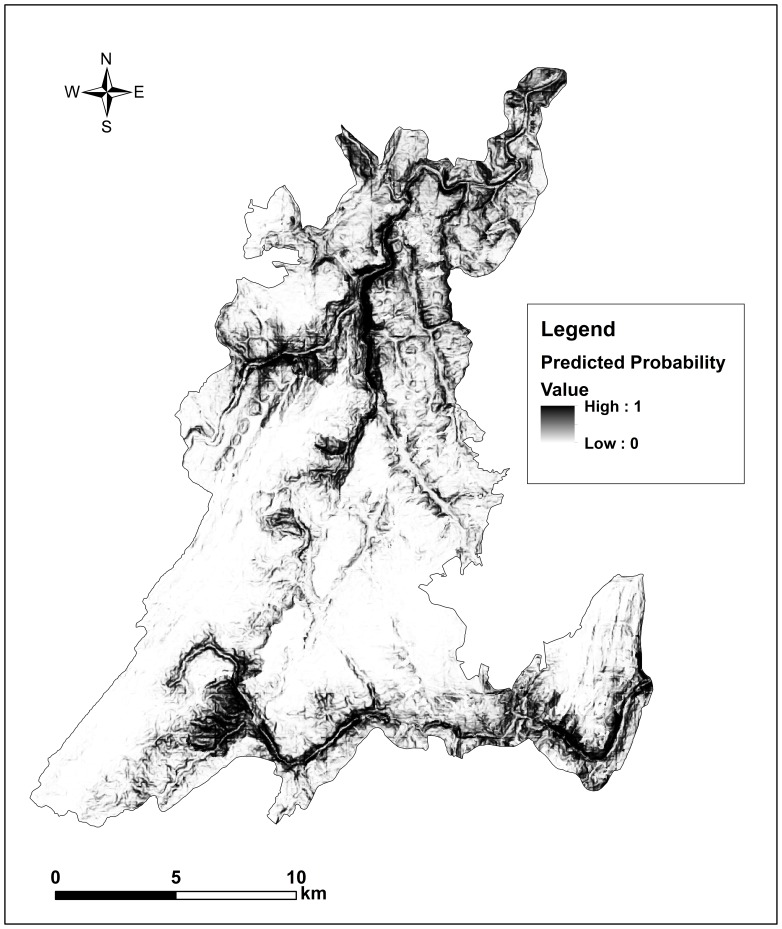
Habitat suitability of Francois’ langur in Mayanghe National Nature Reserve, China based on the use probability predicted by the logistical regression model (**Table 3**). The darker the color, the higher the habitat suitability.

Based on the best prediction model, we estimated the distribution of the habitat suitability using predicted probability of use in this reserve (Figure 3). The average use probability (threshold) was 0.46, and the suitable habitats (probability >0.46) for langurs were mostly distributed in valleys with elevation <1200 m. These habitats covered an area of 7,510 ha and accounted for about 25% of the total reserve area.

## Discussion and Conclusion

Sound management planning requires an understanding of habitat use, identifying where priority habitats are located and determining how species respond to relevant disturbances [30]. Conducting such studies for Francois’ langur, however, was particularly challenging due to the difficult terrain. The current distribution of many endangered species is possibly the result of historical events such as habitat change or factors outside of the reserve including loss of habitat in adjacent areas and large scale climate change [31]. We were not able to include these factors in our predictive models and hence, our study might be biased by those aspects. This study was conducted primarily in the summer. Therefore, we could not rule out the potential use of other vegetation types during other times of the year. Although our transects were not completely random, we did try to cover all sub-units in the reserve and all vegetation types in each sub-unit. The combination of transect and opportunistic point surveys covered almost the entire reserve (see Figure 1), which reduced the possibility of bias [1].

Our overall best model suggested that vegetation type, vegetation coverage, elevation, slope, proximity to water, and distance to paved road and farmland edge affected the probably of habitat use by the langurs. These patterns were consistent with our field observations and the results from univarite analyses between the used and control points. Langurs tended to prefer bamboo forest and mixed conifer-broadleaf forests with higher tree canopy density and vegetation coverage, in areas with lower elevation, steeper slope, and near water and paved roads. The langurs in our study also tended to use evergreen forests more often than available as suggested by previous studies (see [16]). Francois’ langur is a typical arboreal primate and in general prefers bamboo forest and mixed conifer-broadleaf forest as they provide quality food resources [12], [16] and cover for predator avoidance [6].

The langurs in this study tended to maintain a distance from farmlands. Farmlands in this reserve were used for crop and tobacco planting [5]. Crops planted on the farmlands may provide extra food resources for the langurs. Farming in the area is labor intensive with limited machinery use, and the farmers are often present and tending to their crops. The protection of crops by farmers and seasonal fluctuations in crop availability likely limit their potential contribution to the food resources of the langurs [6]. The langurs may visit crops opportunistically when they are in season and farmers or other threats are absent. This might result in our observations that the langurs generally kept a distance from the farmland edges.

Our results showed that Francois’ langur was not only restricted to landscapes with karst topography, limestone cliffs, and caves of tropical and subtropical zones in Asia, but also preferred specific landscape features at the local scale. Francois’ langur was often detected in and around valleys and near water sources, which confirmed the results of previous studies [6], [32]. Additionally, the slope along these rivers was >60 degrees with abundant culverts and karst caves providing ideal roosting sites [6].

Francois’ langur seemed to be found closer to paved roads, which was similar to that found in Yezhong Nature Reserve, Guizhou Province [33]. This was also similar to observations made of the tufted grey langur (*Semnopithecus priam*) in Mudumalai Tiger Reserve, southern India [34]. Langurs may occur close to the road to beg food from tourists [34]. We encountered this situation during our study. However, care must be taken to deal with roads, as it might change the behavior of langurs and introduce additional disturbances. We suggest that long-term monitoring of the effects of roads on the langurs at multiple scales should be initiated to assess their effects accurately.

Many factors may have resulted in the langur population decline [2]. Habitat loss and increased human activities were probably the major factors contributed to this trend at this reserve. China holds the fastest growth of forest plantations in the world in recent years, and the coniferous forests occupied a larger part of these forest plantations [35]. Coniferous forests dominated by Chinese fir (*Cunninghamia lanceolata*) and pine (*Pinus* spp.) expanded from less than 4600 ha before 2000 (Management Bureau of Mayanghe National Nature Reserve, unpublished data) to about 5111 ha (i.e. 16.4% of this reserve) by 2009 with an increase of 11.1%. The area of farmland also increased from 8335 ha in 2005 (Management Bureau of Mayanghe National Nature Reserve, unpublished data) to 9467 ha in 2009 (i.e. 30.4% of this reserve) with an increase of 13.6%, though land conversion in nature reserves is prohibited by the government [36]. The mixed conifer-broadleaf forests and evergreen broadleaf forests preferred by the langurs have been mostly replaced by farmlands and pine plantations (Management Bureau of Mayanghe National Nature Reserve, unpublished data). Tobacco as a cash crop is becoming more popular, and the demand for cultivatable land is generally high in the area due to the karst topography and cultivation patterns, the trend of farmlands expansion is expected to continue in the coming years. Another potentially important factor implicated in langur population decline is logging and fire-wood collection for cooking and heating [5], though logging in reserves is also prohibited by the government [36].

We estimated a total of 7510 ha of suitable habitat remain in the reserve at the time of this study, which equals approximately 25% of the total reserve area. Suitable habitat of langurs is concentrated in valleys and is under threat of increased human activities and population growth. Human population in Yanhe County and surrounding areas, near the reserve, has increased about 8% during the last decade [37]. Tourism has become one of the most economically important industries of Guizhou province [37], [38], and Mayanghe National Nature Reserve has been designated as one of the important tourist destinations [38]. In addition, as in other parts of China, there has been major increase in economic activities and infrastructure development. For example, the economic output (GDP) of Yanhe County has increased about six times in the last decade [39], and two major highways have been constructed, linking Chongqing (a major city in southwest China) to Yanhe County. These activities likely negatively affect the langur population in the area. For instance, we were unable to find langur activity along the upstream areas of the Mayang River, which has ideal habitat features such as low elevation, steep slope and proximate to water, and which used to be the main distribution area of Francois’ langurs in this reserve [40]. However, increased human traffic, including loud diesel boats that run up and down stream, and other disturbances negatively impact this species.

Therefore, it appears that the risks of the disappearance for langurs at this site are increasing. Unless effective conservation strategies and law enforcement are implemented soon to minimize the further habitat deterioration [5] and enhance the habitat restoration, this endangered species may disappear from this reserve. Both the Chinese government and other organizations have now acknowledged the decrease in suitable habitat for the langur in this area, and a combined conservation has been initiated. For instance, the China Biodiversity Conservation Strategy and Action Plan (2011–2013) [41] was recently implemented to target and strengthen the conservation and habitat restoration effort for endangered species, with increased law enforcement in the reserves being considered as one of the most important actions, and our study area was considered as one of the priority areas in the plan. The State Forestry Administration, as well as Fauna & Flora International (FFI), conducted pilot programs to offer more feasible and effective alternatives to basic living requirements for the local communities since the late 1990s. We strongly recommend the protection of areas with high suitability identified by this study, along with adjacent areas, from disturbance and degradation. Some shrubby areas, coniferous forests, and farmlands in or adjacent to suitable habitats should be restored to mixed conifer-broadleaf forests. To reduce the demand for logging in this reserve, it is urgent to disseminate the information about this langur to the local communities. In addition, supplementary energy techniques and other economic incentives should be introduced to prevent traditional firewood collection and other disturbances to the langur habitat in the reserve and surrounding area.
